# Yellow Fever in Non-Human Primates: A Veterinary Guide from a One Health Perspective

**DOI:** 10.3390/vetsci12040339

**Published:** 2025-04-06

**Authors:** Remco A. Nederlof, Tommaso Virgilio, Hendrickus J. J. Stemkens, Luiz C. C. Pereira da Silva, Daniela R. Montagna, Abdussamad M. Abdussamad, John Chipangura, Jaco Bakker

**Affiliations:** 1 Independent Researcher, 2861 XZ Bergambacht, The Netherlands; 2 Institute for Research in Biomedicine, Università della Svizzera Italiana, 6500 Bellinzona, Switzerland; tommaso.virgilio@irb.usi.ch; 3 Stichting Vogelpark Avifauna, 2404 HG Alphen aan den Rijn, The Netherlands; rickstemkens@gmail.com; 4 Institute of Science and Technology in Biomodels, Fiocruz, Rio de Janeiro 21040-900, Brazil; luiz.cavalcanti@fiocruz.br; 5 Institute of Biological Chemistry and Biophysics (UBA-CONICET), Buenos Aires C1428EGA, Argentina; daniela.r.montagna@gmail.com; 6 Faculty of Veterinary Medicine, Bayero University, Kano PMB 3011, Nigeria; amabdussamad.vpb@buk.edu.ng; 7 Faculty of Veterinary Science, University of Pretoria, Onderstepoort 0110, South Africa; john.chipangura@up.ac.za; 8 Animal Science Department, Biomedical Primate Research Centre, 2288 GJ Rijswijk, The Netherlands; bakker@bprc.nl

**Keywords:** One Health, yellow fever, vector-borne, viral disease, neotropical primate, zoonotic, arbovirus, mortality, howler monkey, prevention

## Abstract

Yellow fever (YF) causes severe morbidity and mortality in Africa and South America. It is an arthropod-borne viral disease endemic to tropical regions of Africa and South America. Yellow fever virus (YFV) is transmitted by mosquitoes and frequently affects both non-human primates (NHPs) and humans. Neotropical primates (NTPs) are generally more severely afflicted by YFV than African primates. Asian primates appear not to be susceptible to this disease. Susceptibility varies among NTP species: asymptomatic infections are described in some NTP species, whereas severe epizootic mortality events are described in others. The genus *Alouatta* (howler monkeys) is considered to be the most susceptible among the NTPs. Epizootic events resulting in the death of thousands of NTPs have been recorded in recent history. As a result, YFV poses a threat to the survival of some NTP species. In most cases, NTPs are found dead without showing prior clinical signs. In cases where clinical signs are observed, they are mostly non-specific. Due to their high susceptibility, epizootic events in NTPs are used as epidemiological predictors for human YF outbreaks. YFV infection may be diagnosed by means of virus isolation, reverse transcription polymerase chain reaction, serology, histopathology, or immunohistochemistry. Animals that survive the disease develop neutralizing antibodies to YFV. Currently, no specific treatment is available. Sustained YF control strategies must rely on surveillance and accurate diagnostics to allow for early detection of outbreaks and rapid implementation of control measures. Prophylaxis should be based on a One Health perspective that recognizes the intricate interplay between human health, primate health, and the environment. Vaccines for YF are available, with the human 17DD vaccine effectively preventing disease in primates. However, mitigation strategies continue to rely more and more on vector control, preferably using eco-friendly methods. Climate change and human activities, and their impact on local ecology, are assumed to increase the risk of YF transmission in the next decades.

## Simple Summary

Yellow fever (YF) is a serious viral arthropod-borne disease in Africa and South America, affecting both humans and non-human primates (NHPs). The susceptibility of different NHP species to YF varies greatly, with the largest mortality events reported in howler monkeys (genus *Alouatta*). Neotropical primates (NTPs) often show non-specific clinical signs, if any, before dying. These outbreaks in NTPs may be used to signal potential outbreaks in humans. For these early warning systems to be effective, accurate and reliable diagnostic techniques that work under field settings are essential. There are no YF-specific treatments available, but the human 17DD-vaccine effectively prevents the disease in NHPs. Prophylaxis should be based on a One Health perspective that recognizes the intricate interplay between human health, primate health, and the environment. Consequently, mitigation strategies continue to rely more and more on vector control, preferably using eco-friendly methods. Climate change and human activities, along with impact on local ecology, are assumed to increase the risk of YF transmission in the next decades. This underscores the importance of future research, which should focus on refining YF monitoring systems and developing immunization techniques that are applicable to wild NTP populations.

## 1. Introduction

Yellow fever (YF) is an infectious disease caused by the yellow fever virus (YFV), a member of the family *Flaviviridae*, genus *Orthoflavivirus*. YF is an acute viral hemorrhagic and viscerotropic disease in both human and neotropical primates (NTPs). The term “yellow” refers to the jaundice that is reported in some patients. YFV is a so-called arthropod-borne virus (arbovirus) and is primarily transmitted through the bite of infected mosquitoes [1,2]. The YFV is currently endemic to the tropical regions of Africa and South America, where it is thought to persist in canopy-dwelling mosquitoes and non-human primates (NHPs). Three transmission cycles are described: jungle (sylvatic), intermediate (savannah or rural), and urban [1,3,4,5].

Despite the wide and inexpensive availability of effective vaccines, YFV still poses a major threat to public health in both South America and Africa. This may be due to increasing deforestation, which increases the contact between the canopy-dwelling *Haemagogus* sp. mosquito vector and humans; travel and migration between endemic and epidemic regions; and low immunization coverage in some areas that were previously not considered at-risk for YF [6,7,8]. The World Health Organization (WHO) estimates that there are 200,000 human cases of YF annually across the globe, resulting in 30,000 deaths. The occurrence of YF epizootics depends on environmental factors as well as on the implementation of preventive strategies, and on the geographical and local distribution of mosquitos and NHPs [1,4]. Additionally, different NHP species show dramatic differences in their susceptibility to YF, ranging from asymptomatic infections in African NHPs to highly lethal outbreaks in a range of NTP species (see Section 3.1. Differences in Susceptibility and Clinical Outcomes Among NHP Species) [9,10,11,12,13,14,15,16,17,18]. Due to the susceptibility of some NTPs to the virus, they may function as epidemiological markers, or sentinels, for disease surveillance purposes.

Sustained YF control strategies must rely on surveillance and diagnostics to allow for early detection of outbreaks and rapid implementation of control measures. As the clinical signs of YF resemble those of a wide range of diseases, specific and rapid diagnostics are needed [19,20]. Diagnosis is routinely performed by virus isolation, reverse transcription polymerase chain reaction, serology, histopathology, or immunohistochemistry. Vaccines for YF are available, with the human 17DD vaccine being efficient in preventing the disease in primates (see Section 7.2. Vaccination Strategies for Humans and NHPs in Yellow Fever-Endemic Areas). Prophylactic strategies should be rooted in a One Health perspective that recognizes the intricate interplay between human health, primate health, and the environment [21]. For example, mitigation strategies are progressively evolving toward vector control, preferably using eco-friendly methods. Climate change, human activities, and their impact on local ecology are expected to increase the risk of YF transmission in the next decades (see Section 4. Vector Ecology and Disease Transmission).

This review aims to systematically analyze all the studies about YFV in NHPs, focusing in particular on epidemiology, diagnostics, treatment, and prevention. To identify all the relevant literature, we conducted a search for books, book chapters, and peer-reviewed publications in academic literature databases, such as PubMed and Google Scholar. Additionally, we aim to provide a comprehensive one-health point of view, which may help in controlling YF by acting at different levels, including hosts, vectors, and the environment.

## 2. Epidemiology of Yellow Fever in Non-Human Primates

### 2.1. Geographic Distribution and Prevalence in NHP Populations

**Figure 1:**
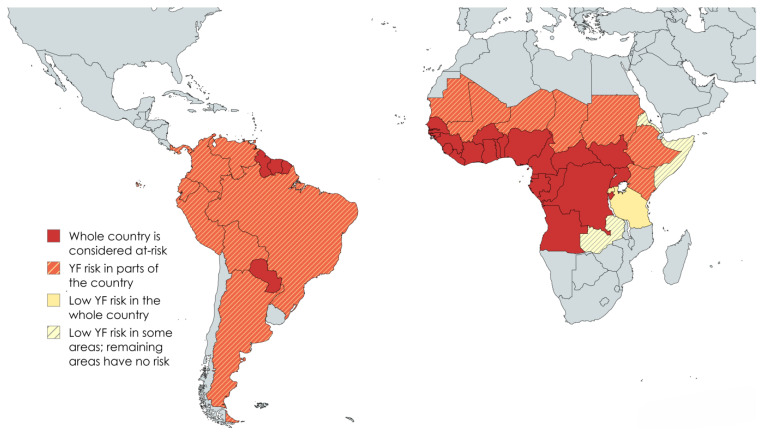
Countries considered at-risk for YFV transmission. The countries indicated on this map are defined as of November 2022 by the World Health Organization (https://www.who.int/publications/m/item/countries-with-risk-of-yellow-fever-transmission-and-countries-requiring-yellow-fever-vaccination-(november-2022), accessed on 20 February 2025) as areas where YF has been reported currently or in the past and vectors and animal reservoirs currently exist.

Yellow fever is an emerging and re-emerging zoonotic disease, periodically causing epizootic outbreaks in the tropical regions of Africa and South America (Figure 1) [1,22,23]. Humans and NHPs are believed to be the major hosts, although antibodies against YFV have also been reported in other species, e.g., rodents, artiodactyls, carnivores, and xenarthras in South America [1,15,24]. Moreover, an older, anecdotal report proposes a potential role for African birds in the maintenance of YFV in Africa based on serological analysis [25]. However, no further literature exists to support a significant role of birds in the maintenance and distribution of YFV. In the Amazon region in Brazil, where YF is considered endemic, the disease seems to follow a cyclic dynamic, causing outbreaks in humans and NTPs at intervals varying between seven and fourteen years [26]. No clear explanation has been provided yet, but it is hypothesized that the severity and persistence of a YF outbreak depends on a complex interplay between vectors, (reservoir) hosts, and the environment [3,27].

### 2.2. Recent Yellow Fever Outbreaks

Yellow fever has been a high-impact, high-threat disease since the 17th century [28]. Whereas the virus does not compromise the survival of primates in Africa, significant illness and population declines in NTPs in South America have been attributed to YF [29,30].

An outbreak in 2008–2009 of sylvatic YF resulted in the death of over 2000 howler monkeys in Brazil [15,31]. Several large YF outbreaks occurred in Brazil between 2016–2018, which are thought to have killed thousands of primates [32,33,34]. Howler monkeys (*Alouatta* sp.) and marmosets (*Callithrix* sp.) were the most frequently affected species during these epizootics. From 2014 to 2018, over 30% of the wild population of golden lion tamarins was lost due to YF [11,12,35]. The most recent YF circulation events in São Paulo occurred in 2023, with confirmed epizootics in NHPs and human cases [36].

## 3. Pathogenesis and Clinical Manifestations

### 3.1. Differences in Susceptibility and Clinical Outcomes Among NHP Species

Whereas African NHPs, including macaques, are reported to experience inapparent infections with low mortality rates [29], the impact of YF on South American NTPs can be tremendous. For example, epizootic YF cases are considered a serious threat to the survival of some wild species such as the northern masked titi monkey (*Callicebus personatus*), golden lion tamarins (*Leontopithecus rosalia*), northern muriqui (*Brachyteles hypoxanthus*), and howler monkeys (black-and-gold, *Alouatta caraya*, and brown, *Alouatta guariba clamitans*) [9,10,11,12,13,14,15,16,17,18].

Based on the number of dead monkeys found during epizootic events, howler monkeys and marmosets are generally assumed to be more susceptible to the disease than capuchin monkeys (*Sapajus sp.)*, titi monkeys (*Callicebus* sp.), squirrel monkeys (*Saimiri* sp.), and spider monkeys (*Ateles* sp.) [14,35]. This contrasts with other reports, where marmosets are described to be less sensitive to YFV infection [34]. However, it must be considered that the reported number of dead and recovered animals is influenced by their proximity to humans, overall population numbers, and species-specific lifestyle and behavior. Therefore, the number of recovered animals may not accurately reflect the susceptibility of a given NHP species. The susceptibility of the NTP species to YFV infection has been investigated by comparisons of naturally infected NTPs with uninfected cohorts. Capuchin and titi monkeys had higher viral loads but lower proportional mortality rates. Marmosets were the least sensitive, showing lower viral loads, lower proportional mortality rates, and no demonstrable YFV antigen or extensive lesions in liver, despite detectable viral RNA [34,35,37,38]. However, such differences have not been associated with the pattern of pathological changes in these animals.

### 3.2. Comparison of Clinical Signs in NHPs and Humans

Humans most commonly experience mild, nonspecific clinical signs with uneventful recovery from infection that may take several weeks up to a few months. Up to 15% of people develop more severe symptoms, and the mortality rate of hospitalized patients is reported to be as high as 30–50% [39,40]. The clinical signs may resemble those of a wide range of diseases. For example, observed jaundice may mimic other viral hemorrhagic diseases, leptospirosis, or viral hepatitis [19,20,41,42]. Therefore, clinical signs are considered to be of little specificity for the diagnosis of YF.

In NTPs, spontaneous acute deaths are most frequently reported during epizootic events [43]. Experimental infections of animals have allowed for the characterization of the clinical and pathological changes and pathogenesis of YFV infection [44,45,46,47,48,49,50,51]. It was demonstrated that YF follows the same course of disease in experimentally infected NHPs as described in humans [48,51,52,53]. The pathophysiology of renal failure, coagulopathy, vascular instability, and shock caused by the YFV is extensively described [40].

In humans, YFV infection is characterized by three stages [28,54,55]. During the incubation period, which lasts 3–6 days, virus can be detected in the blood, and patients may experience headache, fever, vomiting, fatigue, myalgia, and nausea. This is usually followed by remission with abatement of clinical signs for 24–48 h. In some patients, this phase is followed by the return of clinical signs at a more severe level (stage 3). In this stage, the hemorrhagic and hepatic signs of illness occur, along with multi-organ dysfunction. This phase is accompanied by jaundice, from which YF derives its name, vomiting (black vomit or vomito negro, other former names of the disease), and other signs associated with vascular disturbances, such as vascular leakage. Fever, liver dysfunction, renal failure, hypercoagulopathy, and platelet dysfunction may lead to *shock* and subsequent death.

### 3.3. Pathophysiology of Yellow Fever in NHPs

Yellow fever virus elicits two distinct and separate patterns of infection and injury: viscerotropism and neurotropism. Viscerotropism refers to the ability of the YFV to infect and cause damage to visceral organs, including the liver, spleen, heart, and kidneys. Neurotropism, on the other hand, refers to the ability to infect the brain parenchyma and cause encephalitis. The infection and injury of hepatocytes are central to the development of coagulopathies and the resulting hemorrhages reported in NHPs, as hepatocytes are the principal producers of circulating coagulation factors, and massive hepatocellular injury may lead to hemorrhagic diathesis [56,57,58]. YFV has a broad cellular tropism, and recent evidence suggests that extrahepatic targets of infection may contribute to the pathogenesis of severe YF. It has been speculated that decreased clotting factor production alone cannot explain the extent of coagulopathy observed in severe YF. It is proposed that a consumptive coagulopathy, a major component of disseminated intravascular coagulopathy (DIC), occurs as part of the YF pathogenesis [40,44].

### 3.4. Pathological Changes Reported in NHPs

Although (histo)pathological changes associated with YF have been extensively described in humans, there is limited information about these changes in NHPs following natural or experimental exposure to the virus [33,37,38,48,52]. Most experimental studies date from the 1930s [45,46,47,49]. It was demonstrated that YF follows the same course of disease and pathology in experimentally infected NHPs as described in humans [33,34,37,43,49,52,59].

Only howler monkeys have well-documented liver involvement following natural YFV infection. They are described to develop liver failure with massive hepatocellular death. Moreover, massive midzonal hepatocellular necrosis and apoptosis (Councilman body formation), steatosis, liver hemorrhage, hepatic inflammatory mononuclear cell infiltration, acute renal tubular necrosis, and interstitial nephritis have been reported [33,34,37,38,43,49,60].

A recent study identified significant differences in the hepatic histopathological features of YFV infection between NHPs and humans [61,62]. Although fulminant infection has been observed across various South American NHP genera, *Alouatta* spp. appear to develop more severe viral hepatic damage compared to *Callithrix* sp. and *Sapajus* sp.

## 4. Vector Ecology and Disease Transmission

### 4.1. The Roles of Mosquitoes in the Transmission of Yellow Fever Virus

There are three different types of transmission cycles described, all involving vectors from various mosquito genera and vertebrate hosts depending on the geographical location [1,3,4,5,14,23,63,64]. Mosquitoes of the genera *Haemagogus* and *Sabethes* are implicated in South America, and those belonging to the genus *Aedes* in Africa [4,7,20,65,66].

Factors that may favor the spread of the disease and increase the likelihood of large urban outbreaks include more abundant vector populations over a wider geographic area, increasing and accelerating urbanization, low population immunity due to low vaccination rates, and increasing human travel [39,67]. The displacement and fragmentation of NTP populations due to deforestation has been demonstrated to result in higher population densities, often in close proximity to humans [68]. As a result, deforestation may contribute to an increase in YF incidence. It was shown in a YF outbreak that most human infections occurred within an 11 km radius of the finding of an infected NHP, which is in line with the flight range of the primary mosquito vector *Haemagogus* sp. [69].

The absence of YFV in Asia despite the presence of an immunologically naïve population of over 4 billion people while *Aedes* sp. are abundant remains of epidemiological interest [40,70,71,72]. One hypothesis is that prior immunity to related flaviviruses, e.g., dengue or Zika virus, modulates YFV infection and transmission dynamics [71,73].

On the other hand, experimental studies have shown that while the presence of heterotypic flavivirus antibodies does not reduce the risk of YFV infection, dengue-immune individuals who become infected with YF may have a lower likelihood of transmitting the virus to mosquitoes. This suggests that regions not hyperendemic for dengue, such as southern China, Hong Kong, and Singapore, could be vulnerable to YF outbreaks, as the level of population immunity to dengue may not be as extensive as in other parts of Southeast Asia [74].

### 4.2. Transmission Cycles in Sylvatic, Urban, and Transitional Zones

The mosquito vector species live in a variety of habitats; some breed close to human settlements (domestic breeding), others in the jungle (wild breeding), and some in both environments (semi-domestic breeding). All three transmission cycles occur in Africa, whereas only the sylvatic and the urban cycles are described in South America.

(1) Sylvatic or jungle cycle. In the tropical rainforests, primates serve as the primary reservoirs, while humans are incidental hosts. The initial period of infection lasts several days, during which the YFV is present in blood. Wild mosquito species belonging to the *Sabethes* and *Haemogogus* (South America), and *Aedes* (Africa) genera serve as vectors. Because African primates are mostly unaffected by the infection, outbreaks only become apparent once humans are infected [29]. This contrasts with the important role of NTPs as sentinels in South America (see Section 5.1. Role of NHPs as Sentinel Species for Yellow Fever Outbreaks).

(2) Intermediate cycle, sometimes called the savannah cycle or rural cycle. This cycle occurs at the interface between urban and rural or forested land covers and is unique to Africa. In this form of transmission, so-called “semi-domestic” mosquitoes, which may breed both in the wild and close to settlements, infect both humans and primates. Increased human–mosquito contact results in a subsequent increased rate of transmission, and outbreaks may occur at the same time in several villages throughout an area. *Aedes* sp. mosquitoes are implicated in this cycle, causing isolated rural epidemics, which have the potential to trigger larger urban outbreaks [4,5,20,69,75].

(3) Urban or classical cycle. This cycle occurs in areas with high *Aedes* sp. populations, dense human populations, and where people have little to no antibodies against YFV. Under these circumstances, the virus is easily transmitted to large numbers of people by so-called “domestic” infected *Aedes* mosquitoes. In this cycle, humans, and not primates, act as the primary reservoir for the virus. The occurrence of urban YF outbreaks among humans in South America has been significantly reduced by vector control campaigns, vaccination coverage, improved surveillance, and case management. Nevertheless, urban YF outbreaks in South America may still occur, but the probability is low, and transmission is likely to remain highly localized [76].

### 4.3. The Impact of Landscape Modifications in Yellow Fever Epidemiology

Deforestation significantly reduces NHP habitats, forcing them closer to humans; the resulting stress on wildlife can lead to greater susceptibility to disease and may increase the possibility of disease transmission between wildlife and humans [12,33,77,78]. As deforestation and habitat encroachment progress, human populations may encroach more into previously untouched forest areas, increasing contact with YF vectors and the potential for transmission of the virus to humans [79,80].

Anthropogenic changes to the landscape play a crucial role in altering the epidemiologic patterns of infectious diseases, occasionally resulting in cross-species transmission [81]. Ecological studies in epidemiology assessed statistical relationships between YF events and forest fragmentation, indicating that municipalities with a higher potential for virus spread were characterized by increased forest fragmentation. Moreover, the models with stronger empirical support revealed a significant association between forest edge density and the risk of epizootic disease occurrence. They also highlighted the necessity for a minimum threshold of native vegetation cover to limit virus transmission [79,80]. Strategies for forest conservation are necessary for the control and prevention of YF and other zoonotic diseases that can spill over from the fragmented forest remains to populated cities [78,79,80,81].

### 4.4. The Role of Climate Change in Yellow Fever Virus Epidemiology

The possibility of the spread of YFV to non-endemic areas in Mexico, North America, Asia, Europe, and the Caribbean areas is a growing public health concern [82,83,84,85]. Climate change has created conditions favorable for the spreading and infestation of new areas by mosquitoes [8,84,85,86]. A temperature-dependent vector abundance has been proposed, where warmer temperatures may allow mosquitoes to emerge and mature faster [87]. A temperature that is too high, however, may result in larvae not developing into adults, and can even be fatal to the larvae [87,88,89]. Rainfall can also increase mosquito breeding rates by providing stagnant ponds for them to deposit their eggs [8,90].

Computer models indicate that ongoing climate changes will increase the risk of urban and sylvatic YFV transmission, as YFV vectors are expected to spread to novel areas. These models emphasize the need to focus more on the role of vectors and highlight the importance of increased entomological monitoring in areas where populations of often overlooked vectors may thrive because of climate changes [83,84,85,91].

### 4.5. The Role of the Encroachment of Civilization on Natural Habitats

The histories of malaria, dengue, and YFV reveal that, until now, climate changes have rarely been the principal determinants of their prevalence or range; human activities and their impact on local ecology have historically been much more significant [83]. Humans alter the environment at unprecedented rates through habitat destruction, fertilizer usage, nutrient pollution, and the application of herbicides and insecticides. Water-storage jars and drums, cemetery urns, discarded rubber tires, buckets, bromeliads, flowerpots, and other man-made containers can be prolific sources of mosquitoes that would naturally breed in tree holes [83,92,93,94]. Degradation of natural habitats together with reductions of wild animal populations and consequent lack of blood sources for sylvatic mosquito vectors may have accelerated recently, increasing the human biting incidence and the opportunities for vector sharing between NHPs and humans. Besides the degradation of natural environments, the death of NHPs due to YF may have led mosquitoes to feed on humans more frequently, in the absence of other blood meal sources, increasing the risk of human infection [6].

## 5. Zoonotic and One Health Perspectives

### 5.1. Role of NHPs as Sentinel Species for Yellow Fever Outbreaks

Most YF outbreaks in NTPs and humans are reported in the same timeframe, suggesting a connection between the epizootics in these species [15,26,43]. The increase in human cases following the detection of viral circulation in NHPs highlights the critical role of NHP surveillance in the monitoring of YF. This surveillance facilitates the early detection of viral circulation while the virus remains confined to its enzootic cycle, thereby facilitating timely public health interventions [36].

Between 1999 and 2000, after a period of high transmission of sylvatic YF in the Brazilian Midwest region involving record numbers of human and NHP cases during the same period, Brazil began to register the deaths of primates as an early warning sign for the risk of YF [77]. The release of the Epizootics Surveillance System (ESS) for NHPs in 1999 was a significant milestone in the battle against sylvatic YF in Brazil. The system provides timely detection of potential outbreaks by closely monitoring primate health, particularly in regions where the virus had historically posed a significant risk [95].

To this end, howler monkeys are considered excellent natural sentinel species for the early detection of YF epidemics, as they often show acute manifestations of disease with high mortality rates. Moreover, they are widely distributed in South America, allowing for the application of similar protocols in different countries [20,96,97]. A similar ESS cannot be established in Africa, as African primates mostly experience asymptomatic infections [29].

### 5.2. One Health in Surveillance and Control Strategies

Conservation efforts for non-human primates are intrinsically linked to public health considerations regarding YF. The re-emergence of sylvatic YFV is often followed by human YF cases and a loss of biodiversity, and may pose a threat to vulnerable primate populations [12,78,79]. From a One Health perspective, the interconnectivity of human, animal, and environmental health underlies the effective management of YF. Veterinary professionals and biologists play crucial roles in this approach by monitoring the health of NHPs populations, conducting diagnostics, and reporting findings that may alert health officials. Collaboration between veterinary and human health systems is essential for addressing zoonotic diseases like YF. Effective communication and data sharing may improve our understanding of the disease dynamics and allow for more effective implementation of preventive measures. Additionally, understanding the ecological contexts, such as climate and habitat changes, human activities, and vector ecology, is vital for determining the local risks to both primates and humans.

## 6. Surveillance and Monitoring

The clinical diagnosis of YF is problematic because the clinical signs resemble those of a wide range of diseases, including other hemorrhagic viral diseases [19]. In addition, the relatively quick clinical progression of YF in NTPs makes early diagnosis difficult. This is particularly true for wild NTPs, as affected monkeys are often found dead on the ground.

For diagnostics, biological samples should be collected, stored, and transported as soon as possible after death [98]. The prioritized samples are blood, serum, and biopsies of visceral organs, preferably liver, spleen, kidney, heart and lung [41]. Because YFV is primarily transmitted by arthropod vectors, no YFV-specific biosecurity measures are required during sampling. Naturally, basic measures aimed at preventing zoonotic disease transmission of other diseases from carcasses should still be taken, as it is unknown whether an animal harbors zoonotic pathogens other than YFV at the time of death. Local diagnostic laboratories should be consulted about sample storage and transport conditions, as these may vary depending on the diagnostic test used.

YF may be diagnosed by serological detection of IgM antibodies with enzyme-linked immunosorbent assay (ELISA), virus isolation (cell culture), and reverse transcription polymerase chain reaction (RT-PCR) on virus isolation-positive cultures [7,20,65,99]. Histopathological analysis with immunohistochemistry performed on liver sections is considered the gold standard assay for diagnosis by the WHO. Immunohistochemistry may be used to detect YFV antigens in liver, spleen, kidneys, heart, lung or brain tissues [34,41,52,100].

Virus isolation is a viable option for the confirmation of YFV infection. However, the necessity for specialized equipment and appropriately equipped laboratories precludes its use as a first-line diagnostic tool.

Novel real-time RT-PCR assays may discriminate between vaccine- and wild-type YFV strains [65,101]. These techniques are labor-intensive, which may impede early detection of YFV in NHPs, humans, and mosquitoes, delaying the implementation of YF countermeasures due to the need for specialized equipment and skilled technicians.

The loop-mediated isothermal amplification technique (RT-LAMP) is another valuable, rapid, sensitive, and cost-effective alternative. This nucleic acid amplification method works under isothermal conditions, with results visible to the naked eye [102]. However, these assays are currently available only in a certain laboratories.

Another isothermal technique is reverse-transcriptase recombinase polymerase amplification (RPA). This sensitive technique works under even lower temperatures, between 25–42 °C, and may yield results within 15 min. RPA requires few resources, and may therefore be useful in rural or field settings [103].

The use of serological assays is effective after the viremic phase but has clear disadvantages for the detection of active infection. Anti-YFV IgM antibodies develop within a few days after infection and can generally be detected for up to three months, whereas IgG antibodies develop within days after the IgM response and can be detected for years afterward. One should keep in mind that the presence of IgG against YFV in wild NHPs seems to be a relatively common finding, as they can be detected for years post-infection [104]. Moreover, false-negative results may occur in the early infection stage. Consequently, two samples should be taken at least two weeks apart to demonstrate seroconversion or a fourfold increase in the immunoglobulin titer. Moreover, serological cross-reactions constitute major obstacles in achieving confirmed diagnoses in areas of YFV endemicity where other flaviviruses circulate [99,105].

## 7. Prevention and Control

### 7.1. Treatment of Infected NHPs

There is currently no treatment directly targeting YFV in humans or NHPs. Supportive therapy, e.g., fluids, NSAIDs, and other symptomatic treatments, are reported to improve the clinical situation temporarily but cannot eliminate the infection.

Antibodies and immune modulators, e.g., interferon-α, were demonstrated to be effective in primate models when administered before or within a narrow time window after experimental infection. However, these treatments subsequently proved to be ineffective when given after the establishment of YFV infection [106,107,108]. A few antiviral agents, e.g., ribavirin, have been evaluated in primates, but these have not been demonstrated to be efficacious in primate models [109].

Recently, the therapeutic efficacy of YFV-specific monoclonal antibodies (mAbs) isolated from vaccinated humans was tested in rhesus macaques (*Macaca mulatta*). Passive immunization with these potently neutralizing YFV-specific mAbs ameliorated YF disease in the animals, providing a strong rationale for expedited clinical development of this intervention [110].

### 7.2. Vaccination Strategies for Humans and NHPs in Yellow Fever-Endemic Areas

Once the first viral strains were isolated during the outbreak of 1927, it became possible to develop prophylactic vaccines against YFV [28]. A live-attenuated vaccine (YFV-17DD) exists [22,109]. In humans, a single dose is enough to protect an individual for at least 10 years, after which revaccination is recommended [110,111,112].

The literature pertaining to experimental vaccination of primates for human vaccine development has been largely omitted from this review. Although experimental vaccines have successfully been used to confer protection against YF, these vaccines were mostly not marketed [108,113,114,115,116,117]. Current research suggests that human YFV-17DD vaccines are both safe and efficacious in primates [118,119,120,121,122,123]. Vaccination of NTPs in endemic areas with the human YFV-17DD vaccine has emerged as a public health strategy, as it would reduce sylvatic transmission while also preserving endangered susceptible NTP species [122]. In comparison to live-attenuated YFV-17DD, other experimental vaccines, such as an inactivated YF-17DD vaccine, blocked 83% of infections, while a plant-derived recombinant subunit vaccine blocked 50%, indicating variability in efficacy across different vaccine platforms in golden lion tamarins. That study highlighted the need for further preclinical research with larger non-human primate samples and more diverse vaccine formulations to better understand the immune responses and long-term immunity [124].

Vaccination may be considered in zoos in endemic regions, although YFV infections in zoo-housed NTPs are not reported in the peer-reviewed literature [119]. A major challenge remains in the protection of wild NHP populations. With current techniques, it is not feasible to capture and *vaccinate* all animals in their natural habitats. The development of an oral vaccine that could be used in baits, similar to how an oral rabies vaccine has been used in Europe and the USA to control rabies in foxes and raccoons, respectively, could be the next step in the conservation of threatened wild NTPs [125]. Research has been conducted to develop a live vaccine that can be dispersed by arthropod vectors. However, the YF-17D vaccine strain has been demonstrated to replicate poorly in *A. aegypti* due to the presence of a barrier at the midgut basal membrane [126]. Future research may focus on developing live vaccine strains that effectively replicate in vector arthropods for potential use as autodisseminating live virus vaccines.

### 7.3. Vector Control

#### 7.3.1. Chemical Vector Control

A range of chemical vector control strategies exist. For example, chemical larval control may be deployed as a strategy to decrease vector population sizes. Chemical and microbial larvicides, insect growth regulators, and bacterial toxins are available. However, the greatest obstacle to larval control success is the dependency on the ability to detect, access, and eliminate domiciliary breeding sites. This is a challenging and costly task that often results in low coverage, and thus low effectiveness [127,128].

An approach to circumvent the difficulty of locating and treating immature habitats is autodissemination of insecticides [129,130,131,132]. Pilot interventions with pyriproxyfen, an insect growth regulator that affects the morphogenesis, reproduction, and embryogenesis of insects, have yielded promising results for larvicides. Unfortunately, little information exists on the conditions under which it is optimally effective [127,132,133,134,135,136]. The design of new types of dissemination stations and novel formulations may further improve the effectiveness of autodissemination.

Spatial repellents are products designed to release volatile chemicals into an air space, influencing insect behavior to reduce human–vector contact, and thereby reducing pathogen transmission [137,138]. Repellents are already an important prophylactic tool for travelers and populations living in endemic areas of vector-borne diseases. DEET is the best-known example of a safe, broad-spectrum repellent that provides complete protection over a long period [138,139]. The adverse effects of DEET on the environment and other species are relatively low [140,141,142]. However, existing research on the environmental toxicity and human health risks associated with DEET is limited, warranting further investigation [143].

Research on mosquito chemical repellents continues to advance, along with knowledge of mosquito olfaction and behavior, mosquito–host interactions, and chemical structures [144]. Discovery and optimization of spatial repellent compounds and formulated products based on plant terpenoids, which protect against pest arthropods, are preferred [145,146,147,148,149,150,151].

A novel method for controlling mosquitoes is the use of attractive toxic sugar baits (ATSB) [152,153,154,155,156,157,158,159]. This method is based on the requirement for male and female mosquitoes to consume plant-derived sugars for survival. An “attract and kill” approach has been developed using fruit or flower scents as attractants, a sugar solution that acts as a feeding stimulant, and an oral toxin to kill the targeted mosquitoes. A solution of ATSB can either be sprayed on vegetation or suspended in portable bait stations.

Preferably, a structured vector control program should be implemented to prevent insecticide resistance in mosquitoes, as the number of insecticides available to the public is limited [127,160,161,162,163]. The sustainability of this reduction will also depend on methods to avoid novel population increases from the remaining insects, hatching of dried eggs, and migration of mosquitoes from uncontrolled areas. The identification of potential insecticides is difficult, as they need to meet human safety profiles, be environmentally friendly, and confer adequate protection against arthropod vectors. Other challenges are the washing out of material, degradation by UV light, and heat exposure [164,165]. Novel formulations are required to achieve long-lasting, safe, effective release of insecticides under the anticipated use. However, one must consider that the suppression of a mosquito population by any means may encourage invasion and replacement by competitors. It is neither feasible nor desirable to eliminate wild mosquito populations altogether. Hence, efforts should focus on limiting the contact of arthropod vectors with human and NHPs in urban and suburban environments.

#### 7.3.2. Biological Vector Control

The One Health approach advocates for the development of alternative, environmentally friendly vector control methods [166,167]. Data to demonstrate efficacy and public health value are, however, currently lacking. It will be imperative that epidemiological evidence be generated in trials before wider implementation can be recommended.

From this point of view, a technology was developed to kill mosquito larvae with sound waves, which presents a nonchemical and nonbiological alternative to reduce larval populations in drinking water containers or catchments of water [168,169]. This technique has not found widespread application yet.

Entomopathogenic fungi are known for their effectiveness in controlling both larval and adult stages of mosquito populations by damaging and penetrating their cuticle [170,171,172,173,174,175,176,177]. All recent literature suggests this technique is promising. However, currently, this technique cannot compete with the costs and efficacy of chemical insecticides. It may, however, become a more viable option in light of more strict future insecticide regulations.

Among the discussed alternative approaches, the sterile insect technique (SIT) is experiencing rapid development, with numerous pilot trials being conducted worldwide [178,179,180,181,182,183]. SIT is based on the release of sterilized male insects, traditionally employing irradiation, to suppress vector mosquito populations. The SIT induces random lethal dominant mutations in the germ cells, which prevents effective reproduction.

The ability of *Wolbachia* sp. to reduce the transmission potential of *Aedes* sp. mosquitoes for YFV is being developed and widely deployed [184,185,186,187,188,189,190]. *Wolbachia* is a genus of natural intracellular bacterial symbionts found in many insects that is known to alter the reproduction of its host. Two strategies have emerged [190]. The first one releases *Wolbachia* sp. carrier mosquitoes, both male and female, to replace the wild mosquito population, a process driven by cytoplasmic incompatibility that becomes irreversible once a threshold is reached. This suppresses disease transmission mainly by pathogen blocking and frequently requires only a single intervention. The second strategy floods the field population with an exclusively male population of *Wolbachia*-carrying mosquitoes to generate infertile hybrid progeny. In this strategy, transmission suppression depends largely on decreasing the population density of mosquitoes driven by infertility and requires continued mosquito release. *Wolbachia*-mediated sterility can be used in conjunction with the earlier mentioned sterile insect technique to suppress adult *Aedes* sp. mosquitoes [183].

Promising genetics-based approaches are being developed to reduce or prevent the transmission of YFV [191,192,193]. Novel techniques, such as Clustered Regularly Interspaced Short Palindromic Repeats (CRISPR), are facilitating this research [194,195,196]. Genetic control of mosquitoes includes two basic strategies: population suppression and population replacement. The former aims to eliminate mosquito populations while the latter aims to replace wild populations with engineered, pathogen-resistant mosquitoes. Based on the recent advances in genetic engineering, it is anticipated that antiviral transgenic mosquitoes exhibiting gene drive will soon be developed. However, close monitoring in simulated field conditions will be required to demonstrate the efficacy, environmental safety, and utility of such transgenic mosquitoes [197,198]. Gene drive technologies promise to improve human, animal and environmental health, yet also entail potential ecological risks, as theoretically, the *release* of even a few organisms could result in the distribution of potentially harmful *genes* to entire wild populations [199,200].

An example of a genetics-based approach is RNA interference. It was shown that YFV replication in vivo and in vitro can be efficiently inhibited by RNA interference [201,202]. The release of insects carrying a dominant lethal (RIDL) system is another example of a genetics-based approach. This is a genetics-based SIT strategy for *Aedes* mosquitoes. This strategy reduces vector populations through the release of individuals carrying a transgenic construct, which acts on the late larval stage and the pupae to reduce survival [203]. In contrast to both the SIT and *Wolbachia*-based population suppression strategies, eggs must become fertilized for subsequent impact of RIDL technology [191]. The engineered effector gene is homozygous, repressible dominant lethal, and activates its own promoter in a positive feedback loop but can be regulated using an external activator [127,203].

Antivirals may be useful to impede the circulation of arboviruses between arthropods and humans [204,205]. However, no specific antiviral drugs are currently available for the treatment of YF. Recent advances have been made in small molecule anti-arboviral drugs in mammalian and mosquito cells, which block the transmission of YFV [204,205,206]. Tiratricol (triiodothyroacetic acid) has been demonstrated to be a potent YFV inhibitor both in host cells and in animal models [207]. Rifapentine, a macrolactam antibiotic, was demonstrated to have an antiviral effect on YFV infection in cell lines and animal models [208].

#### 7.3.3. Physical Vector Control

Physical measures include the application of mosquito traps, window screens, bed nets and insecticide-treated bed nets and clothing against mosquitoes [137,209,210].

Mosquito traps have been used as effective surveillance tools for decades, but nowadays, mass trapping has been developed to control *Aedes* sp. [211,212,213,214,215,216,217]. For a trap to be an efficient tool for vector species elimination, it must be highly sensitive and specific for a target species. The most effective traps rely on a combination of attractant cues such as light, heat, moisture, carbon dioxide, and synthetic chemicals. Effective mass-trapping depends on trap quality, trap density and geographic coverage, community involvement, and safety. Successful mass trap deployments depend on a high coverage (>80%) of residential areas, pre-intervention and/or parallel source reduction campaigns, participation from a sustainability transitions perspective, and the application of new-generation larger traps (e.g., Autocidal Gravid Ovitrap, AGO; Gravid Aedes Trap, GAT) to outcompete the remaining water-holding containers.

## 8. Policy and Public Health Implications

A One Health approach is imperative for effective YFV management. This approach recognizes the intricate interplay between human health, primate health, and the environment. Key strategies include the implementation of travel advisories, vaccination campaigns, vector control measures, and early detection and reporting of cases. In the One Health approach, the surveillance of epizootics in NTPs plays a fundamental role in the timely deployment of preventive and control measures in human populations [218,219,220,221,222]. Measures to control the spread of the virus and the occurrence of epidemics may be applied as soon as increased mortality rates are observed in wild NTP populations [34,36].

## 9. Research Gaps and Future Directions

### 9.1. Yellow Fever Surveillance Systems

Early surveillance systems focused on monitoring wild NHP health should be further developed, as they have been demonstrated to effectively predict human YF outbreaks [36]. Unfortunately, similar systems cannot be established in Africa, as African NHPs demonstrate resistance to clinical disease [29]. Alternative systems may involve large-scale arthropod vector trapping and analysis to monitor the prevalence of YFV among vector species. Surveillance systems may not only serve to implement timely mitigation measures for human YF outbreaks but may also allow for timely intervention to reduce the mass mortality of nearby vulnerable NHP populations. Surveillance efforts should be coordinated internationally to optimize their effectiveness.

Such surveillance systems heavily rely on accurate and accessible diagnostic techniques. A range of diagnostic methods are available to demonstrate the presence of YFV in samples. However, these techniques are often poorly applicable to field settings in which research and monitoring may be conducted. The RT-LAMP and RPA techniques may constitute viable methods, but are not currently widely available. As a result, future research may focus on the development of cost-effective diagnostic techniques that can be used in field-settings for the early detection of YFV.

### 9.2. International Collaborations for Disease Control and Vaccination Programs

The current YFV vaccines have been demonstrated to be effective in both humans and NHPs. Whereas these are valuable for the protection of susceptible human communities and captive NHPs, no viable immunization strategy currently exists to protect vulnerable wild NHP populations. Future research should focus on the development of live vaccines that may be administered through baits or vectors. By protecting wild NHPs, and thus reducing the size of wild YFV reservoirs, the occurrence of YFV in both NHPs and humans may be reduced. Vaccination campaigns in wild NHPs should be coordinated internationally in order to maximize their impact.

Coordinated efforts should be made to provide YF vaccines to susceptible human populations in at-risk areas. This is of particular importance in Africa, as no early surveillance systems exist here that may guide the early implementation of other preventive measures.

### 9.3. Long-Term Monitoring of Yellow Fever Dynamics

It is evident that the continuing encroachment of civilization upon previously untouched YFV endemic areas, as well as the suggested impacts of climate changes on YFV distribution and epidemiology, will result in an increase in YFV outbreaks in the future. Forest fragmentation should be mitigated, and a minimum threshold of native vegetation cover should be established to limit viral transmission. Further understanding of epidemiological risk factors, such as climate and habitat changes, human activity, and vector ecology is fundamental to the development of accurate epidemiological YF models.

From a One Health perspective, biological vector control strategies are preferred over chemical methods. Nevertheless, novel chemical formulations may be developed that allow for long-lasting, safe, effective release of insecticides under anticipated use. Biological methods are still in early development and cannot be currently recommended. The impacts of genetic or microbial intervention in wild arthropod populations should be studied further before any field applications can be conducted.

Entomological monitoring should be conducted in at-risk areas in conjunction with the monitoring of local NHP populations. Biological monitoring may allow for the recognition of an increased YFV transmission risk before human cases occur and should be used to evaluate the effectiveness of applied mitigation measures.

## Data Availability

Data are available upon reasonable request.
